# Gelseminum

**Published:** 1867-08

**Authors:** Jonathan W. Brooks

**Affiliations:** Chicago


### GELSEMINUM.

EDITOR CHICAGO MEDICAL JOURNAL.—

*My Dear Sir:*—There are credits given, not infrequently, in medical journals, and sometimes regarded as standard works on medicine and therapeutics, to sources which are not properly entitled to them. Such, indeed, may be the fact in regard to the article Gelsiminum, or Yellow Jasmine, at pages 409 and 410 of the United States Dispensatory, twelfth edition, by Prof. G. B. Wood, Philadelphia.

For example, he attributes "*the discovery of its properties, as*

*a febrifuge, to a planter of Mississippi, through the accident of his servant, and that the remedy passed into the hands of irregular practitioners, and was employed by the eclectic physicians before its virtues came to the knowledge of the profession."*

Allow the writer to call attention to the article Polemonion, book iv., chap. ix., in the work on Materia Medica, in 6 books, by Dioscorides, published during the reign of Nero, which is admitted to have been a standard work for 1600 years; and to the article Jasminum, by Gaspard Bauhien, in his work entitled Pinax, published late in the 16th century; and to the article Apiana, in the writings of Dodonæus, entitled Pemptades, published in 30 volumes, early in the 17th century; also to the article Jasminum, by John Quincy, published early in the 18th century; and to the article Jasminum Officinale, by Charles Linnæus, 1743; and to the article Gelseminum, by Motherby, 1765; and to the article Jasminum, of the New Edinburgh Dispensatory, 1794; and another article, by the author of the London Dispensatory, 1811.

Added to the remedial properties enumerated by Dr. Wood, to wit:—as a febrifuge and vermifuge, and as a remedy in rheumatism, chorea, and epilepsy, pneumonia, neuralgia, and gonorrhœa, it was used by the ancients in ulceration of the os uteri, and for fluor albus.

The leaves, flowers, bark, and root, have each in turn been used and tested, within much less than a century, and pronounced by able observers as valueless, compared with other remedial agents which are used in the treatment of these diseases.

As a perfume, repeated cautions are given as to its use, on account of its dangerous narcotic properties, believed to reside in the flowers and in the bark of the root.

The apparent necessity of thus noticing the above article, in the writings of so indefatigable, excellent, and earnest a worker in the cause of medical science, is to be regretted.

*Chicago, June 5th.* JONATHAN W. BROOKS.

